# New tactics in the design of theranostic radiotracers

**DOI:** 10.1038/s44303-024-00027-1

**Published:** 2024-08-02

**Authors:** Cesare Berton, Simon Klingler, Stanislav Prytuliak, Jason P. Holland

**Affiliations:** Department of Chemistry, University of Zurich, Winterthurerstrasse 190, CH-8057 Zurich, Switzerland

**Keywords:** Chemistry, Coordination chemistry, Inorganic chemistry

## Abstract

In the context of molecularly targeted radiotherapy, dosimetry concerns in off-target tissues are a major limitation to the more wide-spread application of radiopharmaceuticals to treat diseases like cancer. Reducing off-target accumulation of radionuclides in background tissues, whilst maintaining high and specific uptake in disease sites and improving the therapeutic window, requires rethinking common radiotracer design concepts. This article explores ways in which innovative radiotracer chemistry (the making and breaking of bonds) is used to modify interactions with the host organism to control excretion profiles and dosimetry at the tissue-specific level.

## Introduction

Nuclear medicine, and especially molecularly targeted radiotherapy, is booming^1^. Recent clinical successes and approval of agents such as ^177^Lu-DOTA-TATE (Lutathera^TM^, NETTER-1 trials) against somatostatin-receptor positive neuroendocrine tumours^2^ and ^177^Lu-PSMA-617 (Pluvicto^TM^, VISION trials) for treating prostate-specific membrane antigen (PSMA) in prostate^3^ cancers, highlight the growing academic and commercial interest in radiotherapeutic compounds as future frontline treatments. Indeed, the latest market analysis^4^ indicated that the global Nuclear Medicine sector was valued at around $10.65 billion USD in 2023 with projections expecting this to surpass $31.44 billion USD by 2033, at a consolidated aggregate growth rate (CAGR) of 11.45% between 2024 and 2033. This growth is driven mostly by North America (United States of America: ~47% share; $4.88 billion USD) where current diagnostic procedures account for 81.4% of the market share – highlighting the huge potential that is likely to be achieved from the emergence of new radiotherapeutic drugs.

**Fig. 1:**
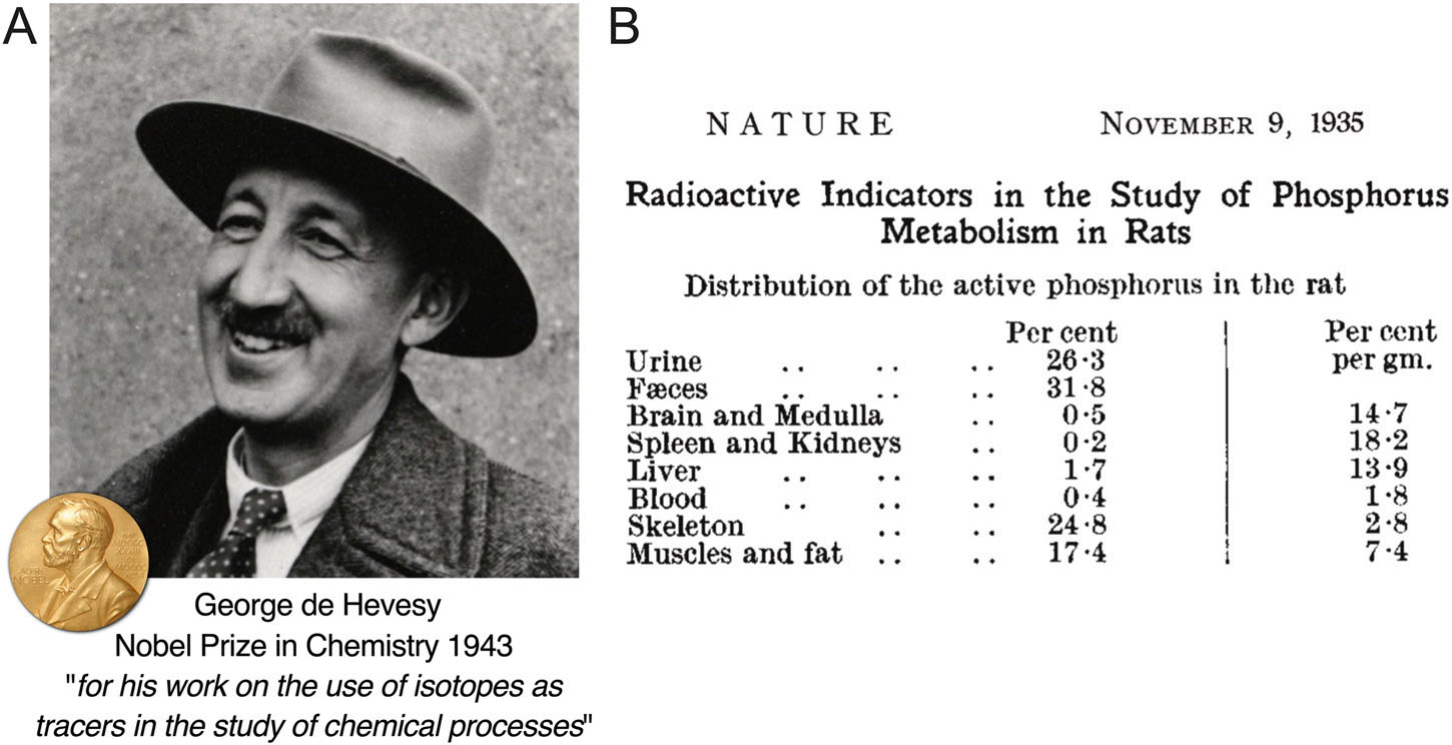
The founding father of radiotracer science. **A** Photograph of Prof. George de Hevesy *circa* 1950 (https://arkiv.dk/en/vis/5941286) who received the Nobel Prize in Chemistry in 1943 for developing the ‘*radiotracer principle*’^8^. **B** An excerpt from a manuscript by Chiewitz and de Hevesy in 1935, reporting one of the earliest examples of a ‘biodistribution study’ using a radionuclide to track the distribution and metabolic fate of [^32^P]NaPO_4_ in rats after ingestion in the food^9^.

Molecularly targeted radiotherapeutic drugs consist of a biologically active vector derivatised with a radiotherapeutic nuclide which is most often either a radiometal ion or a radiohalogen like ^131^I. Other emerging therapeutic radiohalogens of note include the alpha-emitter ^211^At or the Auger electron emitter ^77^Br. The resulting radiolabelled construct is designed to be thermodynamically, kinetically, and metabolically stable in vivo, facilitating the delivery of cytotoxic doses of ionising radiation to the target tissue. The biological vector is typically derived from a small-molecule drug, a modified peptide, a monoclonal antibody (mAb) or related engineered protein fragment that binds to a specific biological marker providing discrimination between normal tissue and diseased cells. The *d*- and *f*-block metal ions of the Periodic Table are a rich source of radiotherapeutic nuclides such as the β-emitters ^47^Sc, ^67^Cu, ^90^Y, ^161^Tb, ^177^Lu, ^186^Re, ^188^Re, and α-emitters like ^149^Tb or ^225^Ac^5–7^. Construction of these metalloradionuclide compounds requires careful consideration of their design in terms of both metal ion coordination chemistry and the nature of the bioconjugate bond formed between the metal ion complex and the biologically active vector. Thanks to the seminal work of George de Hevesy (Fig. 1), who first demonstrated the use of radiolabelled compounds as tracers to track the distribution, uptake, and metabolism of molecules in living systems^8–11^, the starting point for most radiochemists when designing new radiolabelled compounds is the ‘*radiotracer principle*’^12^.

The radiotracer principle has three primary tenets which state that, (i) the concentration of the probe molecule can be measured by quantifying the radioactivity of the sample through time and space, (ii) the radiolabelled compound is either chemically identical to, or a close biomimetic of, the compound under investigation and interacts with the system in a known and reproducible way, and (iii) administration of the radiotracer does not alter or perturb the system under study. Application of the radiotracer principle works very well for diagnostic imaging agents where both the chemical dose and administered activity of the radiolabelled compound are so low that they elicit no pharmacological response. Note, that with respect to the chemical dose of administered radiolabelled mAbs, the United States Federal Drug Administration defines a microdose as <1/100th the dose of a test substance calculated (based on animal toxicological data) to yield a pharmacologic effect. Due to the higher molecular weight of proteins like mAbs (a full IgG_1_ antibody has a nominal molecular weight of approximately 150 kDa), the effective limit for a microdose regimen is set at 30 nanomoles^13^. However, in situations where the system under study is sensitive to even low pico- or femtomolar levels of drug (for example, the potent μ-opioid receptor imaging agent [^11^C]carfentanil^14–16^), and in the context of molecularly targeted radiotherapy where the chemical dose remains sub-pharmacological but higher activity doses are applied to induce ionising radiation damage to tissue, this third tenet breaks down. Exposure of background organs including the liver, spleen, kidneys, bone marrow, and other sensitive tissues like salivary and lacrimal glands to high levels of radiation is often dose limiting with the potential to induce debilitating side-effects for patients.

Contravention of the radiotracer principle by molecularly targeted radiotherapeutics has important consequences that should be considered in the chemical design of new radiolabelled compounds. This article explores the various mechanisms which have emerged to modulate radiopharmaceutical dosimetry, with emphasis on methods that use chemistry to control the distribution, metabolism, excretion, and dosimetry of molecularly targeted radiopharmaceuticals at a tissue-specific level. The reader is referred to the works of ref. ^17^ for further discussion on mechanisms for controlling kidney dosimetry, and the works of various authors on the role of bioconjugate chemistry in radiotracer development^18–22^.

## Classic radiopharmaceutical design

**Fig. 2:**
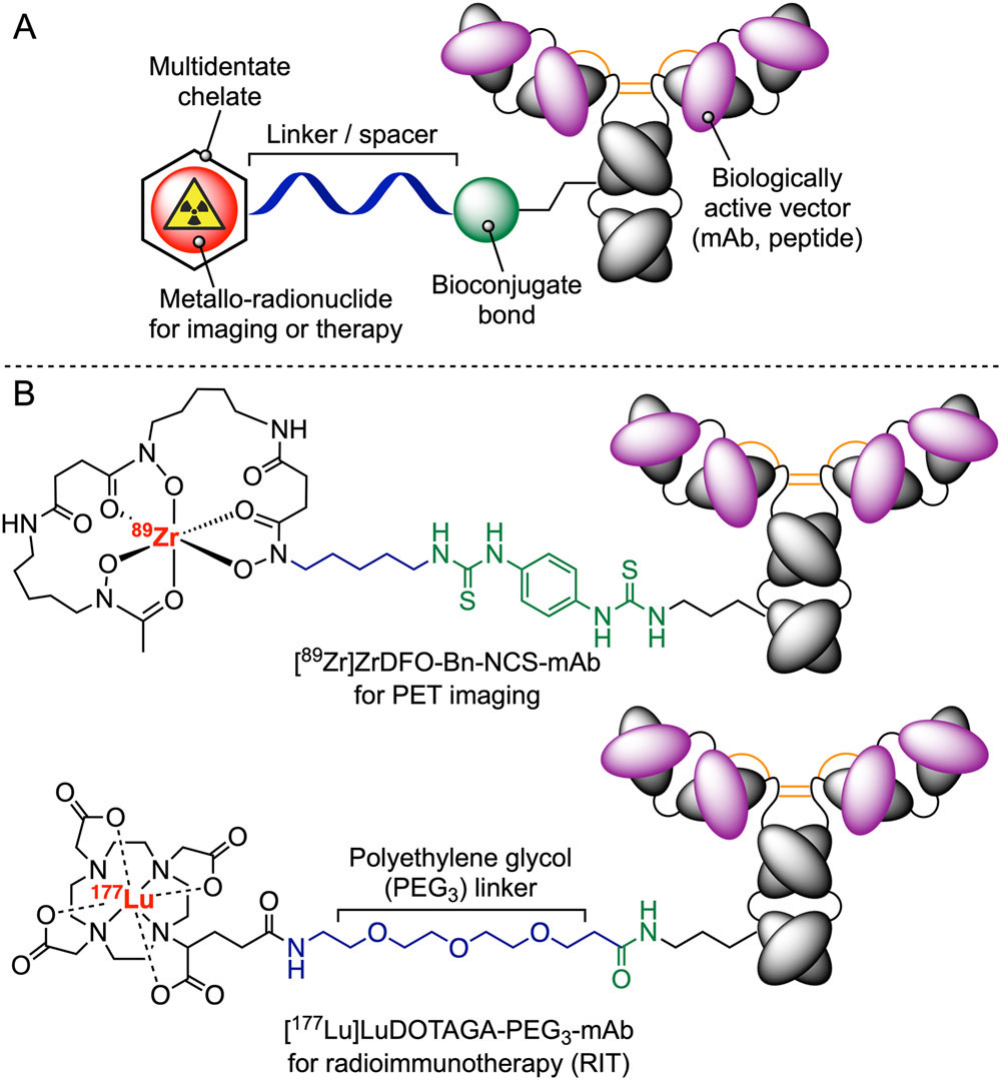
Classic examples of the bifunctional chelate concept in radiotracer design. **A** Schematic of the classic antibody-based radiotracer design employing a covalent modification of the protein structure *via* bioconjugate bond formation (green), a linker or spacer group (blue), and a multidentate chelate (black) that provides thermodynamically and kinetically stable complexation of a specific radiometal ion (red) for applications in diagnostic imaging or radioimmunotherapy (RIT). **B** Two examples of the conventional radiopharmaceutical design showing (top) the structure of a monoclonal IgG_1_ antibody modified with a thiourea bond formed at a surface-exposed lysine residue radiolabelled with ^89^Zr-desferrioxamine B (DFO) complex, and (bottom) a ^177^Lu-radiolabelled mAb employing an octadentate macrocyclic chelate (DOTAGA) and a *tris*-polyethylene glycol (PEG_3_) linker which has been demonstrated to enhance the metabolism and clearance of the radionuclide complex compared with non-PEGylated designs^40,41^. This Fast-Clear^TM^ linker technology is under development at Fusion Pharmaceuticals Inc, Canada (United States Patents: US10093741 and US11191854).

Students and practitioners of metal ion radiochemistry are familiar with the concept of a bifunctional chelator (Fig. 2). In brief, a bifunctional chelator is a molecule displaying two distinct capabilities: (i) coordination of a radiometal ion, and (ii) formation of a bond with a second molecule which is typically a biologically active vector that provides the targeting function to interrogate a specific disease biomarker. Classic radiotracer design, and in particular the use of radiometal ion complexes to label mAbs and proteins, involves several key components (Fig. 2A). First, a suitable radiometal ion is chosen based on the intended application (imaging or radiotherapy)^23^. Metal ion complexation at radiotracer levels requires the use of multidentate chelates which bind to the metal centre with high thermodynamic and kinetic stability, but also shield the metal ion from potential metabolism (e.g., hydrolysis, redox reactions, or ligand exchange processes), avoiding unwanted release and recirculation of the radionuclide. A key objective in chelate design is to complex a radiometal ion selectively and in preference to other naturally occurring metal ions such as Fe^3+^, Cu^2+^, and Zn^2+^ found in high concentrations in the organism. Stable complexation of metal ions is a primary prerequisite since the release of ‘free’ ions like ^89^Zr^4+^ often leads to off-target accumulation in bones and high dose exposure of bone marrow. In addition, rapid and efficient radiolabelling under mild conditions that avoids extremes of temperature or pH is advantageous, and usually essential when labelling mAbs which can denature and lose their biological activity. Second, a suitable bioconjugate handle is chosen that facilitates efficient derivatisation of the protein, ideally without damaging the underlying structure or interfering with the biological activity profile. The development of new bioconjugation methods is an active and fast-paced field of research^18^ where current trends favour chemo-selective and site-specific^24–26^ derivatisation, enzymatic conjugation^27–29^, and our emerging use of photochemical methods^30–34^ that provide access to a wide array of alternative bioconjugate bonds. Examples of mAb-based radiotracers employing classic design principles include ^89^Zr-desferrioxamine (DFO) antibody conjugates for diagnostic immuno-positron emission tomography (PET) (Fig. 2B, top)^35–39^. Recent examples also include the use of polyethylene glycol (PEG) linkers^40,41^ in radioimmunotherapy (RIT), such as the FastClear^TM^ technology in development at Fusion Pharmaceuticals Inc^40^, to modulate the excretion rates of radiotracers in vivo (Fig. 2B, bottom). It is important to note that every feature of the radiotracer design has an impact on the performance in vivo, affecting the biochemical properties including charge, isoelectric point (pI values), lipophilicity, target binding affinity and specificity, non-specific binding profiles, circulation and tissue delivery times, and critically, the potential routes for metabolism and excretion.

This classic design has served the radiochemistry and nuclear medicine communities well during the past >50 years of diagnostic radiotracer development. However, in the era of molecularly targeted radiotherapeutics (or radiotheranostics—which is the combination of both imaging and radiotherapy modalities), rigid adherence to these design principles does not provide the necessary flexibility and sophistication required to address the challenge of controlling (and reducing) off-target radiation dosimetry whilst maintaining high and specific uptake in the disease site. More precisely, to reduce background radiation doses in critical dose limiting organs like the liver, kidneys, spleen, and bones etc, whilst improving the patient tolerance and therapeutic window of molecularly target radiotherapies, we need alternative design principles that leverage differences in biochemistry and metabolism to control excretion of the radionuclide from these tissues and yet avoid loss of the radionuclide from tumours.

## Controlling dosimetry without making or breaking bonds

**Fig. 3:**
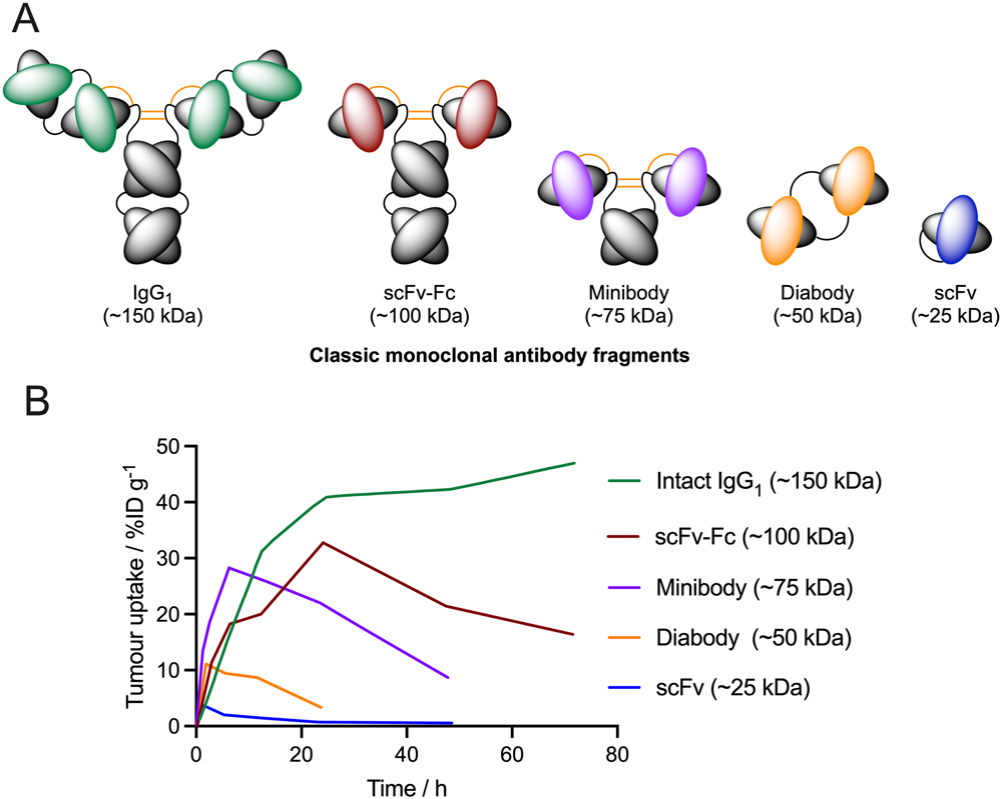
Modulating the global pharmacokinetics of radioimmunoconjugates through protein engineering of antibody size. **A** Schematic structures of conventional monoclonal antibody fragments. **B** Representative plot showing the tumour-associated activity (in percentage injected dose per gram of tissue, %ID g^−1^) *versus* time (in hours) post-radiotracer administration (figure composed based on the work of Wu and Senter^48^ referring to experimental data acquired from radioiodinated monoclonal antibody fragments)^47^.

The problem of drug toxicity in off-target organs is not new and many researchers have sought ways to modulate drug and radiotracer pharmacokinetics^42,43^. Indeed, optimisation of small-molecule drugs^44^ and antibody therapeutics^45^ in ways that reduce off-target toxicity is a classic problem in medicinal chemistry. Most work has focused on reducing the dose to kidneys^17^. Simplistic methods include modulating the size of a protein which has a dramatic influence on the residence times in blood pool circulation, and on trapping in tissue compartments (Fig. 3). Pioneering studies from Anna Wu and co-workers^46–51^ showed that tumour uptake, blood pool circulation, and global residence time of the activity in mouse models can be modulated by using engineered mAb fragments with varying size and amino-acid sequence. Proteins below ~40 kDa in hydrodynamic size are cleared rapidly from circulation, which decreases tumour uptake. Blood pool clearance of these small proteins is accompanied by rapid glomerular filtration and renal excretion into urine. While modification of the radiotracer size provides some measure of control over the distribution and elimination of the radiotracer, the use of smaller proteins and peptides, as well as monovalent mAb variants also induced a dramatic decrease in peak tumour uptake and retention (Fig. 3B). In the ideal scenario, tumour uptake should remain high, with the radionuclide sequestered in the disease foci after delivery, but with either low accumulation or rapid metabolism and excretion of the activity from background tissues. While changing the size of the biological vector provides one way of selecting the baseline pharmacokinetic profile of a radiotracer, altering size alone does not facilitate tissue-specific control of metabolism that will be required of future molecularly targeted radiotherapeutics to reduce dosimetry in background organs.

On the opposite side of radiotracer development from proteins are small-molecule or peptide-based radiopharmaceuticals, which often exhibit rapid clearance from the blood pool. Short circulation times typically reduces the overall peak tumour uptake and retention, which imposes a challenge to delivering sufficiently high doses of therapeutic ionising radiation to affect a pharmacological response. To tackle this problem, researchers have sought to increase circulation times by modifying the chemical structures of small-molecule or peptide-based radiotracers with protein (albumin) binding tags^17^. The concept is to enhance radiotracer retention times in circulation by inducing reversible and loosely bound non-covalent interactions with endogenous globular proteins found in the blood. In this context, the primary mechanism is to induce weak interactions of the radiotracer with human serum albumin (HSA) which is the most abundant protein in human blood found in very high concentrations of ~35–50 mg mL^−1^^52^. Indeed, direct radiolabelling of HSA leads to reasonable PET image contrast and tumour accumulation of ~5%ID g^−1^ due to the enhanced permeability and retention mechanism, which appears to be relatively invariant across a range of preclinical tumour models in rodents^53^. Examples of radiotracers that have been modified with albumin binding units include radiolabelled folate^54^, PSMA^55^, Exendin-4 ligands^56^ and small Designed Ankyrin Repeat Proteins^57,58^. Experimental data on these systems highlights the varying degree of success of this HSA binding strategy on modulating activity in the kidney. Other examples of HSA-binding tags include the use of Evans blue^59^ and recently the introduction of ibuprofen moieties^60^ to modulate PSMA pharmacokinetics. However, as with alteration of radiotracer size for protein-based compounds, the addition of HSA-binding tags does not facilitate tissue-specific control over pharmacokinetics, especially in terms of retaining high tumour uptake and selectivity reducing activity accumulation in background organs. In addition, we note that in contrast to the discussion points that follow, these HSA-binding strategies do not involve the making or breaking of covalent bonds.

Other mechanisms of modulating radiotracer pharmacokinetics include the pre-administration or co-administration of a blocking agent that either prevents or reduces binding of a radiotracer to a particular site—exploiting endogenous differences in target biomarker densities in vivo. An example is the use of the antifolate pemetrexed to reduce kidney uptake of folate-based radiotracers^61^. Alternatively, post-administration of a competitor can be used to displace the radiotracer after initial binding has occurred, potentially facilitating more rapid clearance. Finally, in the domain of antibody-based radiotracers and ADCs, another strategy to modulate circulation times, tumour uptake, and excretion profiles is to use protein engineering methods to manipulate the FcRn binding of full-length mAbs^62^. Whilst successful in modulating some aspects of radiotracer pharmacokinetics, these approaches lack our desired chemical control over tissue specific metabolism in background organs, and are not discussed further.

## Chemical methods for controlling radiotracer dosimetry

**Fig. 4:**
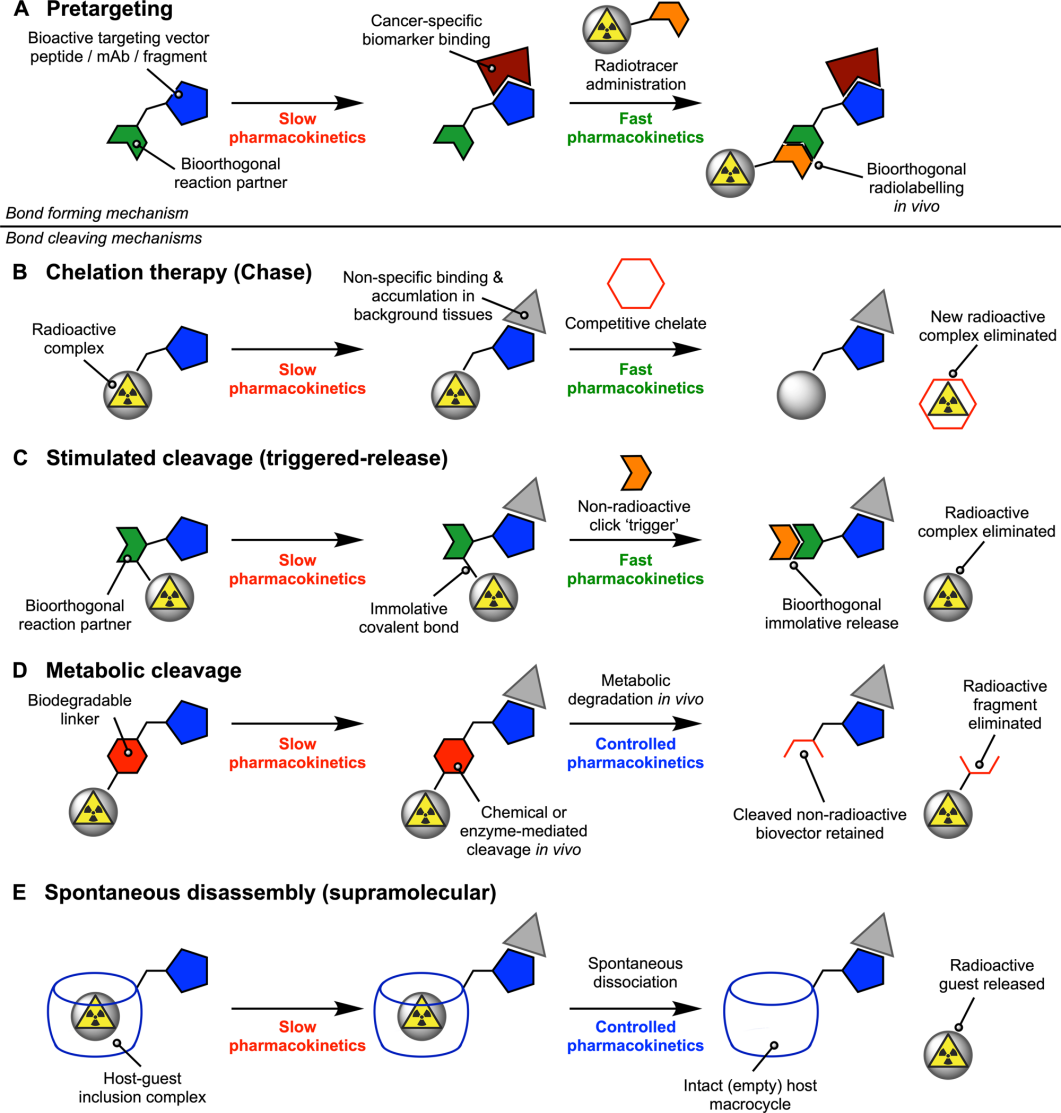
Five conceptually distinct mechanisms that use chemistry to modulate radionuclide dosimetry in vivo. **A** Pretargeting involving pre-administration of a non-radioactive component with slow pharmacokinetics followed by administration of a rapid-action small-molecule radiotracer after a suitable time delay that allows blood-pool and non-specific background clearance for the first agent. Note that mechanisms (**B**–**E**) focus on eliminating radioactivity from non-target tissues. **B** Chelation therapy (or radioactive chase experiments) whereby administration of a classic radiopharmaceutical with slow pharmacokinetics is followed by administration of a competitive binding agent that can (in theory) extract and eliminate the radionuclide. **C** Controlled metabolic cleavage which harnesses endogenous catabolism in vivo to break a bond and release a small-molecule radiolabelled fragment which is eliminated rapidly from the body. **D** Stimulated or chemically-triggered radionuclide (or fragment) release whereby after initial radiotracer delivery, administration of a non-radioactive bioorthogonal partner induces bond cleavage and immolative release of the radionuclide complex. **E** Controlled supramolecular disassembly of a molecular inclusion complex or mechanical bond^72^.

This section introduces the key concept of using chemistry, and specifically the making or breaking of bonds in vivo, to modulate radiotracer pharmacokinetics and control dosimetry. We focus our discussion on the use of mAb-based radiotracers for RIT but the concepts can be generalised and applied to other radiopharmaceutical constructs including small-molecule and peptide-based agents. At least 5 distinct mechanisms that use sophisticated chemistry to modulate radiotracer dosimetry in vivo have emerged (Fig. 4). The most common methods include pretargeting strategies, chelation therapy (chase experiments), or metabolic cleavage *via* endogenous chemical or enzymatic pathways for radiotracer processing^63–66^.

Pretargeting (Fig. 4A) involves the use of fast bioorthogonal reactions to assemble the radiolabelled mAb in vivo^67^. In the most common iteration of pretargeting, the functionalised but non-radioactive mAb which exhibits slow pharmacokinetics and tumour targeting occurs first. After optimum tumour uptake of the functionalised mAb is attained (typically occurring at 24–72 h after administration in preclinical mouse models using full-sized mAbs at ~150 kDa), a radiolabelled small-molecule counterpart is administered. The small, radiolabelled compound distributes rapidly through the body and undergoes an irreversible, bioorthogonal binding to the accessible mAb component. In the absence of a reaction with the bioorthogonal mAb counterpart, the radiotracer is designed to be rapidly eliminated from the blood pool and (usually) excreted *via* the kidneys. Notably, this pretargeting approach is essentially the only established mechanism that involves a bond formation process for modulating radiotracer dosimetry, whereas the other mechanisms presented in Fig. 4B–E involve bond breaking. However, a major problem with implementation of this pretargeting strategy in the clinic is the heterogeneity of drug uptake in different patients and across different disease lesions—defining the optimum time delay between non-radioactive mAb administration and the injection of the small-radiolabelled counterpart is not trivial and is very likely to be patient-specific. Nevertheless, a prominent example of successful pretargeted radioimmunotherapy in preclinical models is provided by the work of ref. ^68^. They demonstrated a dose-dependent therapeutic response in an SW1222 colorectal carcinoma model in mice using pre-administration of the *trans*-cyclooctene-mAb, huA33-TCO, followed 72 h later by administration of the tetrazine derivative, [^67^Cu]Cu-MeCOSar-Tz.

Chelation therapy is based on the competitive complexation of the radioactive metal ion with a small ligand (Fig. 4B). Chelation therapy can also be combined with more sophisticated three-step pretargeting approaches as described by ref. ^67^. The concept stems from the clinical practice of using metal ion binding chelates like ethylenediamine tetraacetic acid to extract and eliminate heavy metal ion toxicants from the body. After waiting for optimum distribution of a radiolabelled mAb, a small-molecule chelating ligand (chase agent) with a rapid pharmacokinetic profile is administered. In theory, if the chase agent has higher affinity for the radiometal ion than the chelator located on the targeting vector, transchelation can occur and a more rapid excretion profile of the small metal ion complex compared with the parented radiotracer can improve off-target dosimetry. In practice, this mechanism is extremely difficult to implement because most radiotracers and metal ion complexes have been explicitly designed to exhibit extremely high thermodynamic and kinetic stability in vivo. Transchelation challenge assays are commonly used during the development phase of a new radiotracer which typically reveal high stability of the radiotracer construct and negligible or slow exchange rates in vitro. If the thermodynamics are in favour of transchelation, then the process could be accelerated by administration of high doses of chase agent. Nonetheless, other complications associated with chelate toxicity and leaching or depletion of essential metal ions from the body may occur, which limit the more wide-spread use of this mechanism of radioactive dosimetry control. However, one application in which chelation therapy is potentially useful is in the elimination of daughter radionuclides that are formed after alpha (or beta) decay chains involving heavy radiometal ions like ^225^Ac. For ^225^Ac-based radiotracers, the release of radioactive daughter nuclides can lead to recirculation of activity in currently unknown chemical forms. These daughter radionuclides increase dose exposure in critical organs including the gastrointestinal tract and are potentially lethal (supported by unpublished experimental data from author JPH)^69^. Successful complexation and rapid clearance of daughter radionuclides will be essential to facilitate the administration of higher doses of the parent (tumour targeted) ^225^Ac-radiotracers.

Robillard and co-workers have used the principles of bioorthogonal click chemistry^70^ to develop an antibody-based ‘click-to-release’ strategy for delivering cytotoxic drugs^71^ or triggering the stimulated excretion of a radiolabelled fragment by using a chemically-responsive cleavage mechanism (Fig. 4C). This advanced method takes advantage of the bioorthogonal chemoselectivity and rapid kinetics of the inverse-electron-demand Diels−Alder (IEDDA) reaction between *trans*-cyclooctene (TCO) and tetrazine derivatives to stimulate the disassembly of a radiotracer in vivo. In contrast with the pretargeting route, the elegance of this ‘excretion on demand’ approach is that the high tumour uptake inherent to the radiolabelled-mAb is retained and the excretion process can be initiated at essentially any time by administration of the non-radioactive tetrazine trigger agent.

The final two mechanisms involve metabolic cleavage using chemistry which is endogenous to the patient (Fig. 4D) and the emerging area of using supramolecular (host-guest) chemistry in biomedical applications (Fig. 4E). The use of cleavable linkers that are responsive to chemical or enzymatic reaction in vivo is arguably the most robust and versatile of the mechanism presented. Further examples are discussed in detail in the section below. Supramolecular systems including host-guest cage complexes and mechanically interlocked molecules (MIMs) present new opportunities for controlling dosimetry in radiotracer design^72–87^. The key feature of supramolecular design is the use of non-covalent interactions to either encapsulate a radionuclide species inside a cavity^72^, or to bind a radiolabelled molecule to a biologically active vector without direct covalent bond formation^84^. By optimising the binding affinity and selectivity of a host-guest system under different external stimuli such as changes in tissue pH or redox potential, it is possible to tune the rate of supramolecular disassembly (Fig. 4E). Other approaches include the combination of metabolic cleavage with controlled disassembly of a MIMs like a rotaxane. Examples of the latter include the experimental studies from Papot and co-workers on the design of enzyme responsive rotaxanes^88,89^ for cytotoxic drug release^90^, the use of cleavable macrocycles for controlled disassembly^91^, as well as our recent work on rotaxane-based supramolecular radiotracers^84–87^.

## Structure-based tactics to control pharmacokinetics

**Fig. 5:**
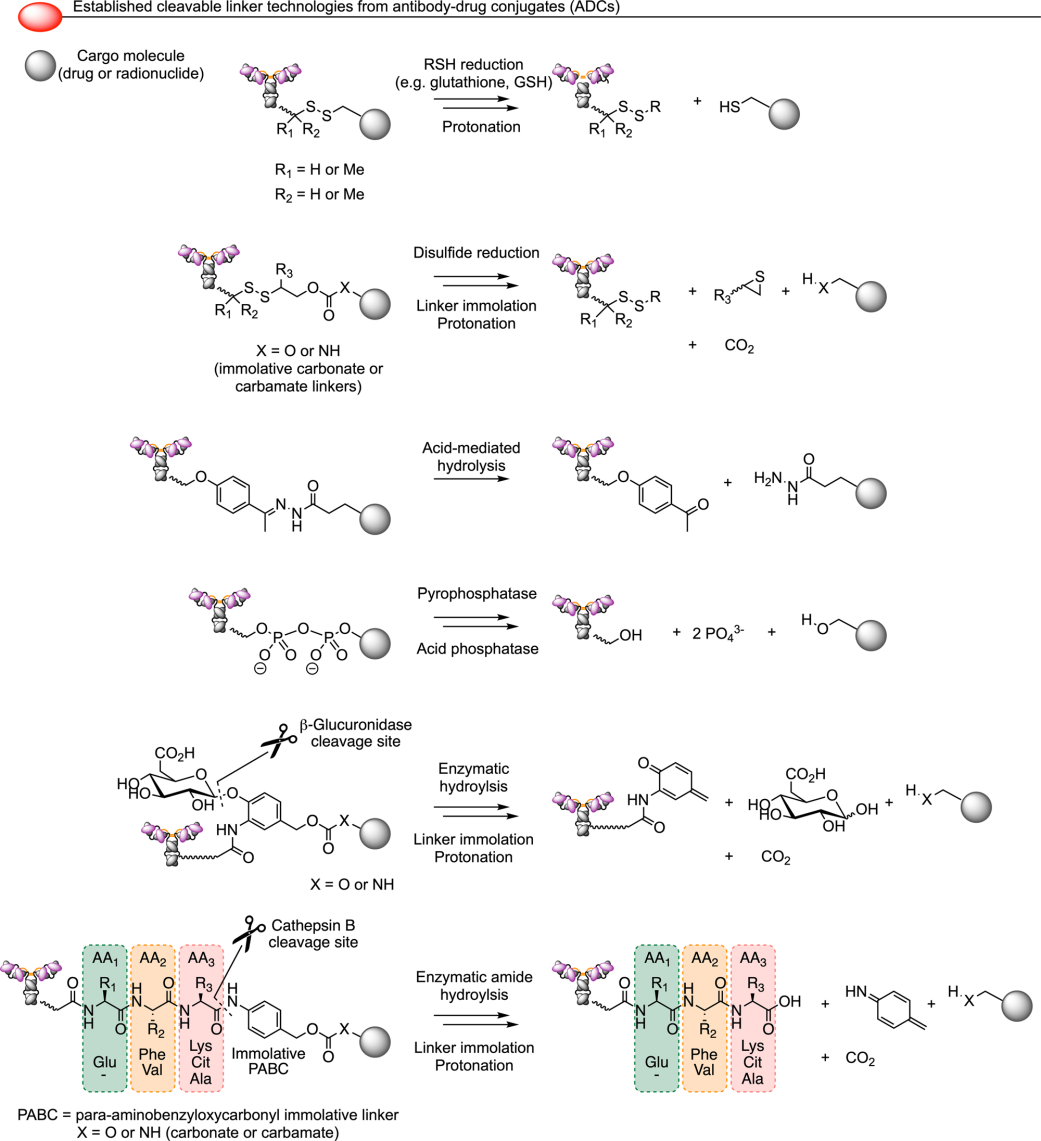
Summary of selected classes of cleavable linkers^92,93^ used in antibody-drug conjugate technologies that could potentially be applied to cleavable radiotracer design via changing the cargo molecule. (Top-to-bottom) A reducible disulfide linker, a disulfide combined with a chemically labile carbonate or carbamate, an acid labile hydrazide linker, an enzymatically-labile pyrophosphate linker, a glycoside linker cleavable by β-glucuronidase followed by linker immolation, and a di- or tripeptide linker hydrolysed by cathepsin B followed by PABC degradation leading to cargo release.

Cleavable linker technologies are a well-established^92,93^ and active area of research^94–98^ in antibody-drug conjugate (ADC) designs (Fig. 5). Most ADCs use immolative linkers that upon chemical or enzymatic cleavage lead to spontaneous degradation and release of an active small-molecule or peptide-based pharmaceutical compound^92,93^. Examples include disulfide linkages which can be reductively cleaved using glutathione (GSH) or undergo sulfide exchange followed by linker immolation and release of the active drug. An alternative chemical strategy involves hydrolysis of acid-labile functional groups like hydrazides.

Several enzyme-mediated linker cleavage methods have also been developed. For example, pyrophosphate linkers hydrolyse via a two-step process involving the dual action of pyrophosphatases (also known as diphosphatases) and acid phosphatases (also known as phosphomonoesterases) to release phosphate anions and the free drug. β-Glucuronic acid has been installed as a trigger for drug release in ADC design stimulated by the hydrolytic action of β-glucuronidase. In addition, a variety of cathepsin-cleavable di- and tripeptide sequences including valine-citrulline (Val-Cit) and glutamic acid adaptations (Glu-Val-Cit)^99^ have been installed adjacent to the *para*-aminobenzyloxycarbonyl (PABC) functional group, that through the loss of a CO_2_ molecule facilitates the release of drugs bearing an alcohol or amine group.

**Fig. 6:**
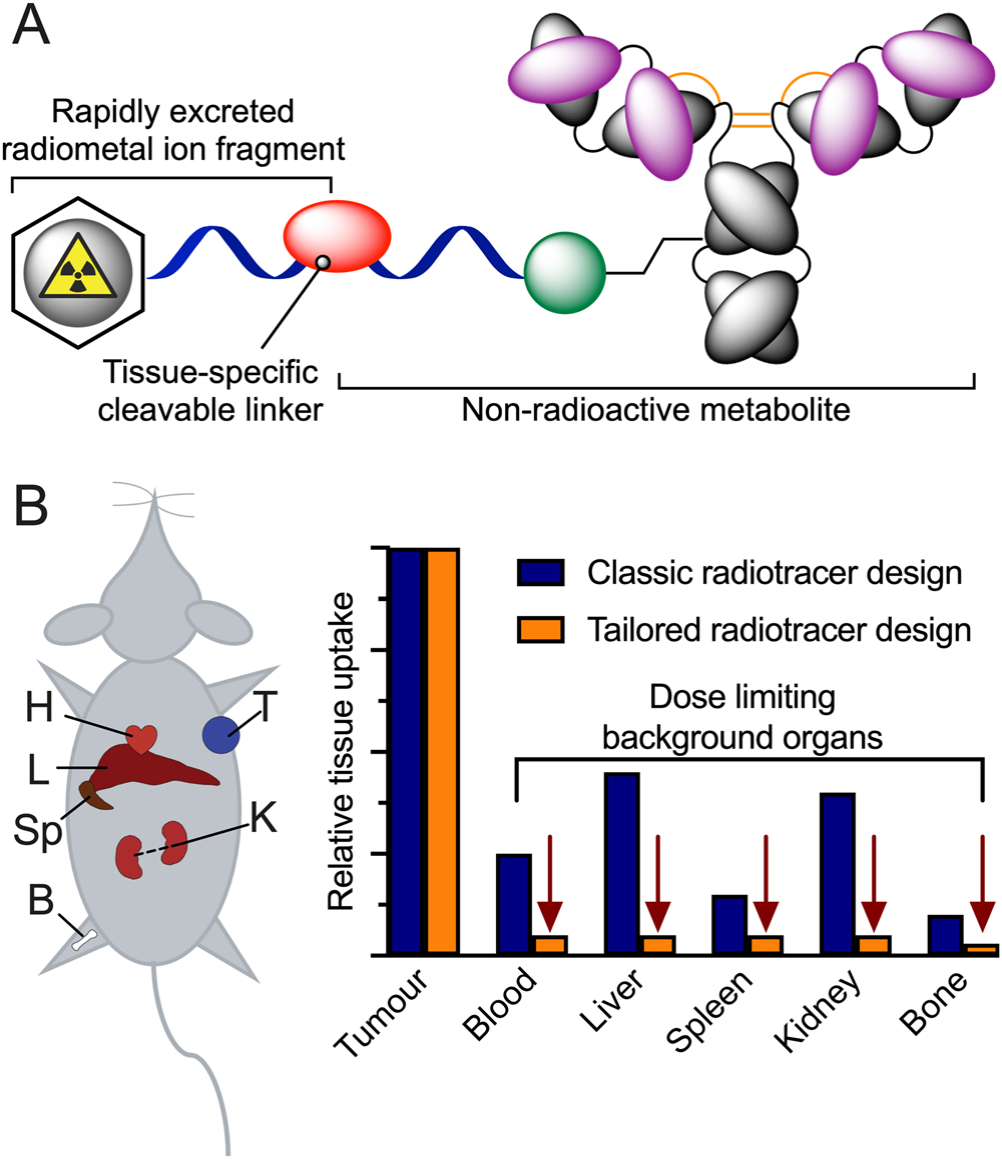
A new design principle for molecularly targeted radio(immuno)therapy agents that draws inspiration from the cleavable linkers used in antibody-drug conjugates (ADCs). **A** Installation of a metabolically cleavable group (red) and/or the use of alternative bioconjugation chemistries (green) that facilitate tissue-specific metabolism in background organs, and rapid (ideally renal) excretion of a stable, radiolabelled small-molecule fragment. **B** Graphical illustration of the objective of retaining high tumour uptake and specificity whilst reducing the dosimetry burden experienced by dose limiting background organs including, but not restricted to: K = kidneys, L = liver, H = heart/blood pool, Sp = spleen, B = bone/marrow.

Taking inspiration from ADC technologies, we propose that for molecularly targeted radiotherapeutics, the classic picture of a bifunctional chelate (Fig. 2A) should be modified to incorporate alternative bioconjugation groups and/or cleavable linkers (Fig. 6A). The objective is to use chemical design to facilitate tissue-specific metabolism. In doing so it is important to retain high tumour uptake, specificity and retention of the radionuclide, but also to achieve rapid clearance of a stable, radiolabelled metabolite from background organs that would otherwise limit dosimetry and reduce the therapeutic window (Fig. 6B). Key design criteria include, (i) thermodynamically and kinetically stable radionuclide complexation that prevents dissociation of the metal ion, (ii) efficient, reproducible and high-yielding bioconjugate bond formation, ideally under mild conditions that avoids denaturation or aggregation of the biological vector, (iii) chemo- and potentially regioselective functionalisation of the biologically active vector, (iv) controlled stoichiometry (chelate-to-protein ratio), and (v) installation of a chemically or enzymatically labile group between the radionuclide complex and the biologically active vector that permits tissue-selective metabolism in vivo.

Harnessing metabolism, rather than attempting to avoid it altogether, is a conceptual step that leads to many additional questions that go beyond established preclinical testing and validation programmes for current radiopharmaceuticals. For instance, the rates of chemical or enzymatic degradation are likely to be dependent on the chemical structure of the molecule. If cleavage is too slow, then elimination of the radiometabolite may not occur on a time scale that would facilitate dosimetric control. Similarly, for enzyme-mediated mechanisms, the cleavage residue must be recognised and accessible to the enzyme active site, and the enzyme expression profile should facilitate metabolism in background tissue but ideally not in the tumour or diseased site. On the other hand, for cleavage systems like the use of Val-Cit linkers coupled to the action of cathepsin B, which is present in the blood, it is also crucial to measure metabolism rates in serum. The experiment in whole serum benchmarks the degradation kinetics in vitro where release rates must be balanced with the time scale required for the biovector to achieve optimum uptake in the target tissue. If cleavage rates are too fast (and especially those mechanisms that involve cleavage in circulation), it may lead to reduced tumour targeting. With these considerations in mind, new assays will be required to measure and characterise the metabolic mechanisms, the cleavage rates, and tissue-specific elimination rates of new cleavable radiotracer designs.

## Cleavable linkers in radiotracer design

**Fig. 7:**
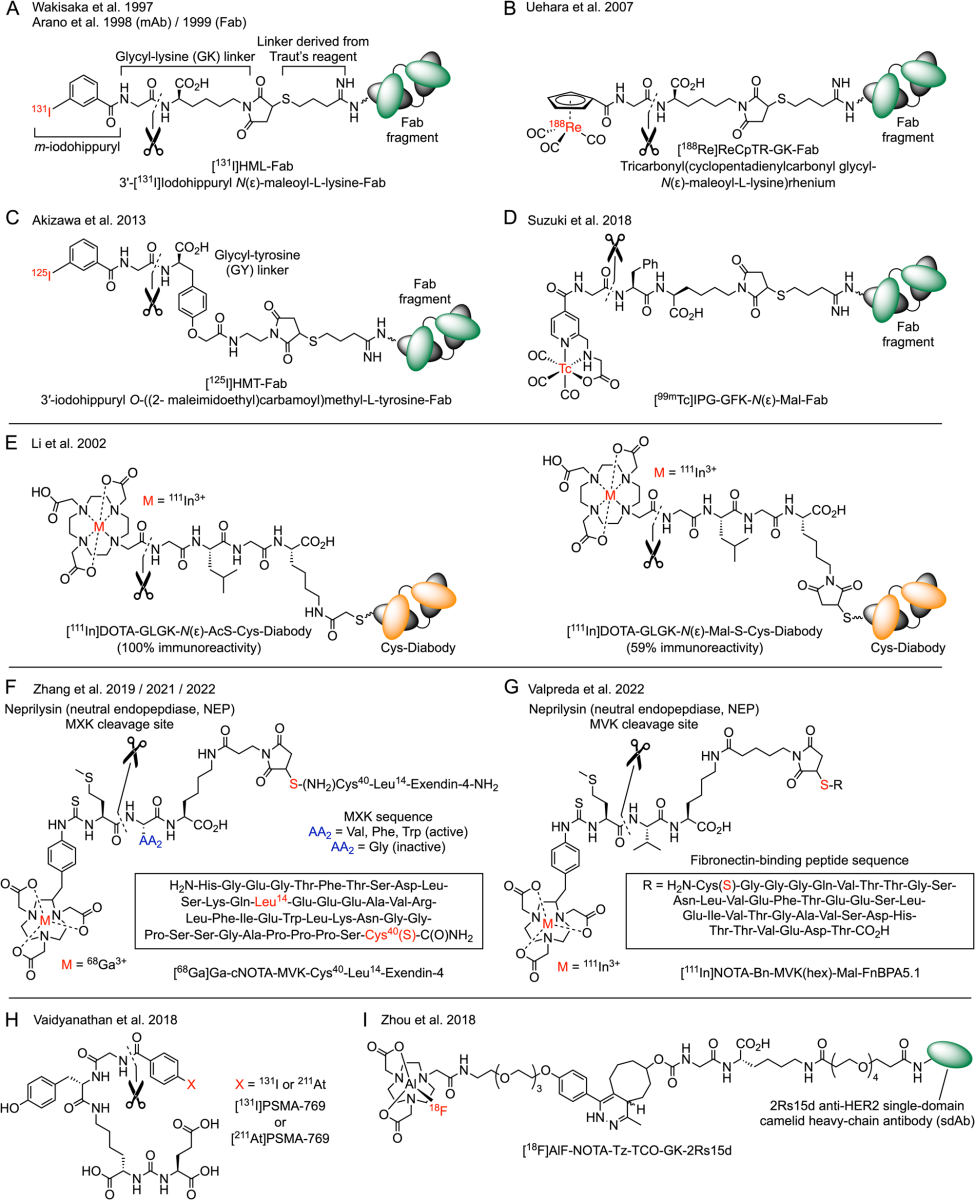
Prominent examples of radiolabelled compounds whose structure includes a cleavable linker designed for hydrolysis in the kidney by brush border enzymes like carboxypeptidase M and neprilysin (neutral endopeptidase, NEP). **A** An early example of a radioiodinated *meta*-iodohippuryl-glycyl-lysine linker^100–103,105^. **B** ^188^Re-radiometal ion complexes featuring the Gly-Lys cleavable linker^104,106^. **C** Installation of a cleavable Gly-Tyr linker to a radioiodinated-Fab radiotracer^108^. **D** A ^99m^Tc(I)-tricarbonyl-Fab radiotracer^109^ for SPECT imaging. **E** An ^111^In-radiolabelled diabody with a cleavable GLGK tetrapeptide^113^. **F** A ^68^Ga-radiolabeled Exendin-4 radiotracer with a cleavable MXK peptide for tuneable hydrolysis by NEP^114^. **G** A fibronectin-binding peptide radiolabelled with ^111^In^3+^ ions *via* a cleavable MVK peptide^115^. **H** A cleavable radiohalogenated (^131^I or ^211^At) derivative of a PSMA-targeting vector with a hydrolysable glycine linker. **I** An Al^18^F-radiotracer with a tetrazine-TCO conjugation, a cleavable GK linker and classic amide functionalisation on a bioactive single-domain antibody (sdAb)^122^. For further examples see the references^116–118^.

Naturally, radiochemists have already used metabolically cleavable linkers in radiotracer design, particularly involving attempts to reduce kidney dosimetry. From the mid-1990s, Yasushi Arano and co-workers performed trail-blazing work on the use of cleavable linkers to improve the renal clearance of protein-based radiotracers (Fig. 7)^100–109^. Initial studies were motivated by the need to reduce kidney exposure to radioiodinated-Fab fragments and studies concentrated on the use of 3′-[^131/125^I]iodohippuryl *N*(ε)-maleoyl-L-lysine ([^131/125^I]HML, Fig. 7A)^100–103,105^. The radioiodination process utilised a two-step functionalisation to attach a *meta*-iodohippuryl group *via* Traut’s reagent (2-iminothiolane) to create a free sulfhydryl group which facilitated bioconjugate bond formation via Michael addition of the thiolate to a radiolabelling agent bearing a maleimide group. The cleavable linker was composed of a glycine-lysine (Gly-Lys) dipeptide which undergoes hydrolysis of the amide bond mediated by membrane-bound carboxypeptidase M, a brush border enzyme located in the single-cell layer of epithelial cells surrounding the lumen of kidney tubules^110^. Experiments demonstrated significant kidney clearance with an approximately 4-fold reduction in kidney activity when compared against direct (non-cleavable) iodination of the Fab fragments^102^. Related studies from the same group^103,105^ and others^111^ have expanded upon this approach, where reports also include the use of ^188^Re-radiometal complexes as potential radiotherapeutic agents (Fig. 7B)^104,106^.

Akizawa et al.^108^ explored an alternative cleavable glycine-tyrosine (GY) linker, functionalised on the phenolic *O*-atom, where the use of inhibitor studies found that the amide bond between the glycine and tyrosine residues undergoes enzyme-mediate hydrolysis by neutral endopeptidase (neprilysin, NEP), carboxypeptidases, and dipeptidases (Fig. 7C). Further developments led to a ^99m^Tc-Fab radiotracer for single-photon emission tomography (SPECT) imaging (Fig. 7D) which features a cleavable tripeptide Gly-Phe-Lys (GFK)^109^. The amide bond between the Gly and Phe residues was highly susceptible to hydrolysis by NEP, partially cleaved by carboxypeptidase B and angiotensin converting enzyme, but not affected by dipeptidyl peptidase. Also in 2018, ref. ^112^ reported preclinical studies comparing the kidney retention and tumour-to-kidney ratios of two cleavable ^67^Ga-labelled Fab fragments, [^67^Ga]GaNOTA-MVK-Fab and [^67^Ga]GaNOTA-MI-Fab, against that observed for a non-cleavable control [^67^Ga]GaNOTA-Fab. Use of the Met-Val-Lys (MVK) linker, which is cleaved by NEP, was found to give a 4-fold and 7-fold higher tumour-to-kidney ratio at 3 h post-injection, with comparable overall tumour uptake compared with that of the cleavable Met-Ile (MI) construct, [^67^Ga]GaNOTA-MI-Fab, and the control [^67^Ga]GaNOTA-Fab, respectively.

Notable studies also include the work of ref. ^113^ who in 2002 reported an ^111^In-radiolabelled diabody featuring a cleavable tetrapeptide Gly-Leu-Gly-Lys utilising two different bioconjugation groups (GLGK, Fig. 7E). More recently, the tripeptide unit MVK has received attention from several research teams^114–120^ with prominent examples provided by the work of refs. ^119,120^ who optimised the cleavage kinetics of a ^68^Ga-radiolabelled Exendin-4 by screening the central amino acid (MXK, where X = Val, Phe or Trp, Fig. 7F)^114^ and ref. ^115^ who also used the MVK linker in the design of fibronectin-binding radiotracers to control kidney activity (Fig. 7G).

Two further examples of radiotracers with cleavable linkers include the work on glycyl-functionalised PSMA by ref. ^121^ (Fig. 7H) and the use of an ^18^F-radiolabelled Al-NOTA complex coupled to an anti-human epidermal growth-factor receptor 2 (HER2) single-domain camelid heavy-chain antibody 2Rs15d by Zhou et al. (Fig. 7I)^122^. This latter example also utilised the original GK linker.

Finally, in 2024, Zhang et al.^123^ reported a first-in-human study of the use of a cleavable MVK modified [^68^Ga]GaNOTA-MVK-Z_HER2:2891_ anti-HER2 affibody, confirming that this enzyme-mediated clearance strategy can effectively reduce renal radioactivity accumulation in humans. This strategy is also expected to reduce the renal radiation burden of peptide and small protein-based radiotracers.

## Supramolecular chemistry in radiopharmaceutical design

**Fig. 8:**
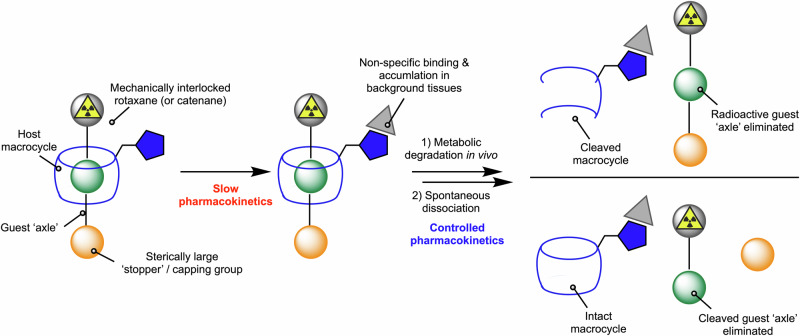
A supramolecular approach for controlling tissue-specific processing of radiotracers. This method is a combination of both metabolically induced bond cleavage and supramolecular disassembly (see Fig. 4D, E, respectively). The schematic radiotracer is composed of a mechanically interlocked rotaxane (or alternatively, a catenane) featuring non-covalent interactions between the macrocyclic host (blue rings) and the guest ‘axle’ molecules. Bulky axle capping agents which act as stoppers to prevent disassembly of the mechanical bond can be tune to include a combination of radiometal complexes, fluorophores, cytotoxic drugs. Metabolic cleavage of a covalent bond in either the host macrocycle (top right) or axle (bottom right) breaks the mechanical bond leading to the release and rapid renal elimination of a small-molecule radiolabelled fragment^85^.

Supramolecular chemistry has been defined as either ‘chemistry beyond the molecule’ or ‘the chemistry of non-covalent interactions’. In this discipline, compounds include host-guest complexes that display reversible binding of one or more guest molecules inside molecular cavities. Further classes of supramolecular compounds also include MIMs like rotaxanes and catenanes which feature mechanical bonds where two (or more) non-covalently bound components can only be separated by breaking a covalent bond^124^. In recent years, several groups^78,88–90,125–130^ from the organic, materials, and radiochemistry fields have begun to explore the potential of using supramolecular chemistry for biomedical applications and this nascent topic has been reviewed^73–76^. Radiochemists including the groups of Roger Alberto^77^, Steve Archibald^72^, Angela Casini^78–83^, and our team^84–87^ have used molecular cages or rotaxanes as platforms for developing Supramolecular Agents as Radio-Theranostic drugs (SMARTdrugs). The synthesis is very challenging, but these supramolecular adducts hold high potential to be a disruptive technology in radiotracer design. For instance, spontaneous disassembly of molecular inclusion complexes (Fig. 4E*vide supra*) or a combined metabolic degradation preceding supramolecular disassembly (Fig. 8) are new mechanisms that can be adapted for modulating radiotracer pharmacokinetics.

**Fig. 9:**
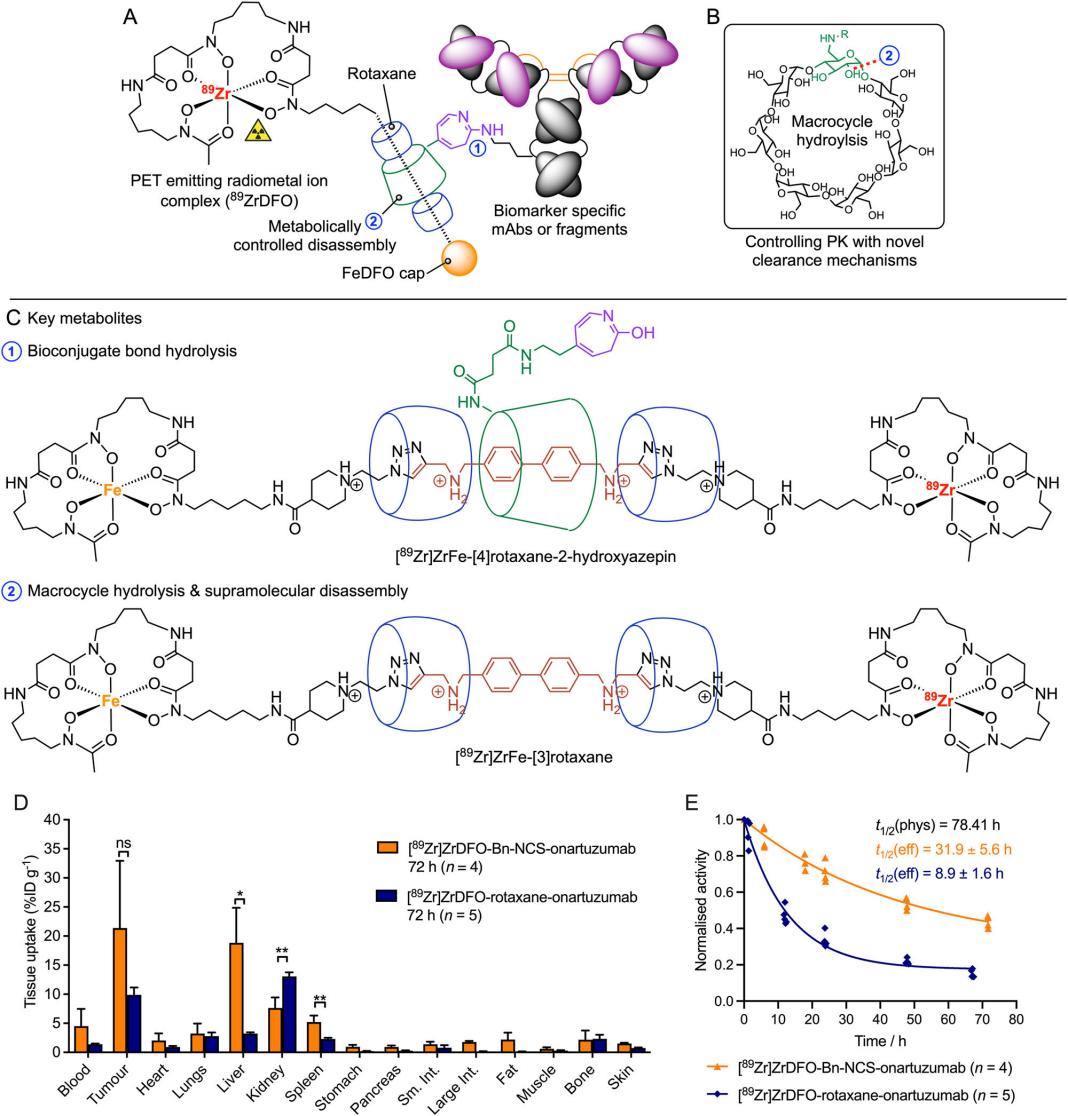
Examples of supramolecular radiotracers constructed using molecularly-interlocked rotaxanes. Head-to-head comparison of the supramolecular rotaxane-based radiotracer [^89^Zr]ZrDFO-rotaxane-onartuzumab^85^ versus conventional [^89^Zr]ZrDFO-Bn-NCS-onartuzumab synthesised via the standard protocol by Vosjan et al.^35^ which is used in the clinical preparation of many 89Zr-mAbs (see Fig. 2B for the molecular structure of the control compound). **A** Schematic representation of the rotaxane-based radiotracer featuring a non-covalent mechanically-interlocked bond between the radiolabelled guest axle and the host macrocycle which is attached via a covalent bond to the mAb or mAb-fragment using our photoradiosynthesis methods^30–34^. **B** A metabolically labile 1,4-glycosidic bond in the functionalised host β-CD macrocycle, hydrolysis of which breaks the mechanical bond of the rotaxane. **C** Potential hydrolysis of (1) the novel bioconjugate bond leading to rapid renal excretion of a supramolecular [4]rotaxane metabolite or (2) supramolecular disassembly to eliminate a radiolabelled fragment. **D** Comparison of the biodistribution of the supramolecular [^89^Zr]ZrDFO-rotaxane-onartuzumab versus conventional [^89^Zr]ZrDFO-Bn-NCS-onartuzumab at 72 h post-intravenous administration in female athymic nude mice bearing subcutaneous MKN-45 gastrointestinal adenocarcinoma tumours, and (**E**) the experimentally measured effective half-life of the two radiotracers showing a clear difference in the distribution and excretion rates.

We reported the first experimental example of using rotaxanes to modulate radiotracer pharmacokinetics in vivo in 2023 (Fig. 9)^84,85^. The complex system includes a β-cyclodextrin (β-CD) as the main host macrocycle which is functionalised with a photochemically active group for light-induced conjugation to lysine residues on antibodies^30–34^. Notably, the rotaxane radiotracer can degrade to release small-radiolabelled rotaxane metabolites in two ways: first, through hydrolysis of the 2-amino-azepin bond to the protein releasing the [4]heterorotaxane [^89^Zr]ZrFe-[4]-rotaxane-2-hydroxazepin; second, through hydrolysis of the 1,4-glycosidic bond in the β-CD host which induces supramolecular disassembly and release of the hetero-[^89^Zr]ZrFe-[3]rotaxane (Fig. 9C). Independent synthesis, characterisation and in vivo studies demonstrated that both radiometabolites are partially cleared from the body via renal excretion (Fig. 9D). These novel pathways for radiotracer metabolism led to an overall effective half-life *t*_1/2_(eff) of 8.9 ± 1.6 h for [^89^Zr]ZrDFO-rotaxane-onartuzumab which was significantly shorter than that observed for the non-cleavable control [^89^Zr]ZrDFO-Bn-NCS-mAb^35^ (*t*_1/2_(eff) = 31.9 ± 5.6 h Fig. 9E, and structure shown in Fig. 2B). Much work remains before these concepts can be adapted more widely in mainstream radiotracer design. Despite the early stage of research, supramolecular radiotracers provide novel design strategies which will add value to the growing repertoire of methods that can be employed to tailor uptake, metabolism, and dosimetry.

## Dosimetry estimates and experimental design for molecularly targeted radiotherapeutics

In this final section, we address the challenges associated with designing molecularly targeted radiotherapy experiments in animal models. Specifically, if one aims to perform a therapy study in a tumour model, it is extremely difficult to select an appropriate activity dose a priori. Pilot experiments or prior knowledge of the model system are required to establish the pharmacokinetic profile of the radiotracer and estimate the maximum or peak tumour-associated uptake (*φ*_max_, phi-maximum) expressed in standard units of percentage injected dose per gram (%ID g^−1^) as measured from biodistribution or temporal imaging studies. In designing appropriate radiotherapy experiments the initial priority is to identify, (i) if the tumour model is sensitive to ionising radiation exposure, and (ii) the threshold dose required to achieve a measurable physiological or pharmacodynamic response to the molecular radiotherapeutic in the treatment group when compared against an appropriate control group. For instance, if the administered activity dose is too high, the tumour might respond but there is a high likelihood that adverse, dose-limiting effects may also occur in radiation sensitive background organs such as the liver, kidneys, spleen, bone/bone marrow or other tissues. It appears frequent in preclinical radiotherapeutic studies to administer high activity doses (sometimes exceeding 100 MBq per mouse) without performing routine controls over blood biochemistry, cellular composition, and pathological investigations of organ function. In addition, because the radionuclide half-lives and the other decay characteristics are not identical between different nuclides, e.g., ^177^Lu *versus* ^161^Tb, a comparison of the potential therapeutic effects of the same radiotracer labelled with different nuclides cannot be interpreted directly from studies that seek to compared equivalent administered activity doses (in MBq). Rather, head-to-head comparisons require administering the same chemical dose, but with an adjusted administered activity (and hence molar activity) to ensure that the total tissue absorbed (energy) dose (in grey [Gy], equivalent to SI units of J kg^−1^) is comparable between radiotracers with different radionuclides. This is not trivial to estimate with accuracy.

To facilitate the prediction of tissue absorbed dose for any given combination of radionuclide, radiotracer pharmacokinetics, and tissue (threshold) sensitivity to ionising radiation, we created a free web application, *DoseItRight*^©^ (https://doseitright.streamlit.app/). The application is not intended to replace full dosimetry software which are used for estimating human dosimetry based on animal data (see Carter and Zanzonico for an excellent overview of this topic^131^) but rather to help guide scientists in selecting the most appropriate activity dose to administer in an animal model and to design suitable controlled studies. A step-by-step guide to the operation of the *DoseItRight*^©^ application is given the electronic supporting information (Figs. S1–S8). Limitations of the model are that it does not account for heterogeneity in tissue physiology, biochemistry, and radiotracer distribution, and crossfire or bystander effects are omitted. The model also assumes that for each atom that decays all energy is deposited uniformly within the tissue and that the absorbed dose neglects contributions from γ-rays (future updates will expand the capabilities of the *DoseItRight*^©^ tool). The application requires the following input parameters, which for any given combination of radiotracer and animal model can be reliably estimated from pilot imaging studies:

- A biphasic pharmacokinetic profile is generated through user-defined half-life times *t*_*i*_ for the process *i* associated with either uptake (*i* = 1) or elimination (*i* = 2) of the radiotracer from the tissue. Values of *t*_1_ and *t*_2_ can be freely selected so that the pharmacokinetic profile provides a close approximation to the experimentally observed situation. Similarly, *k*_*i*_ is the associated decay constant where ${k}_{i}={\mathrm{ln}}\left(2\right)/{t}_{i}$ for the uptake or elimination step. We note that most researchers have a good understanding of the pharmacokinetics of their radiotracers and these data can be reliably measured from pilot imaging or biodistribution studies.
- A peak tissue uptake, (*φ*_max_ /%ID g^−1^).
- An administered activity dose (*A*/MBq).
- Selection of a radionuclide (nuclear data are used from MIRD).

**Fig. 10:**
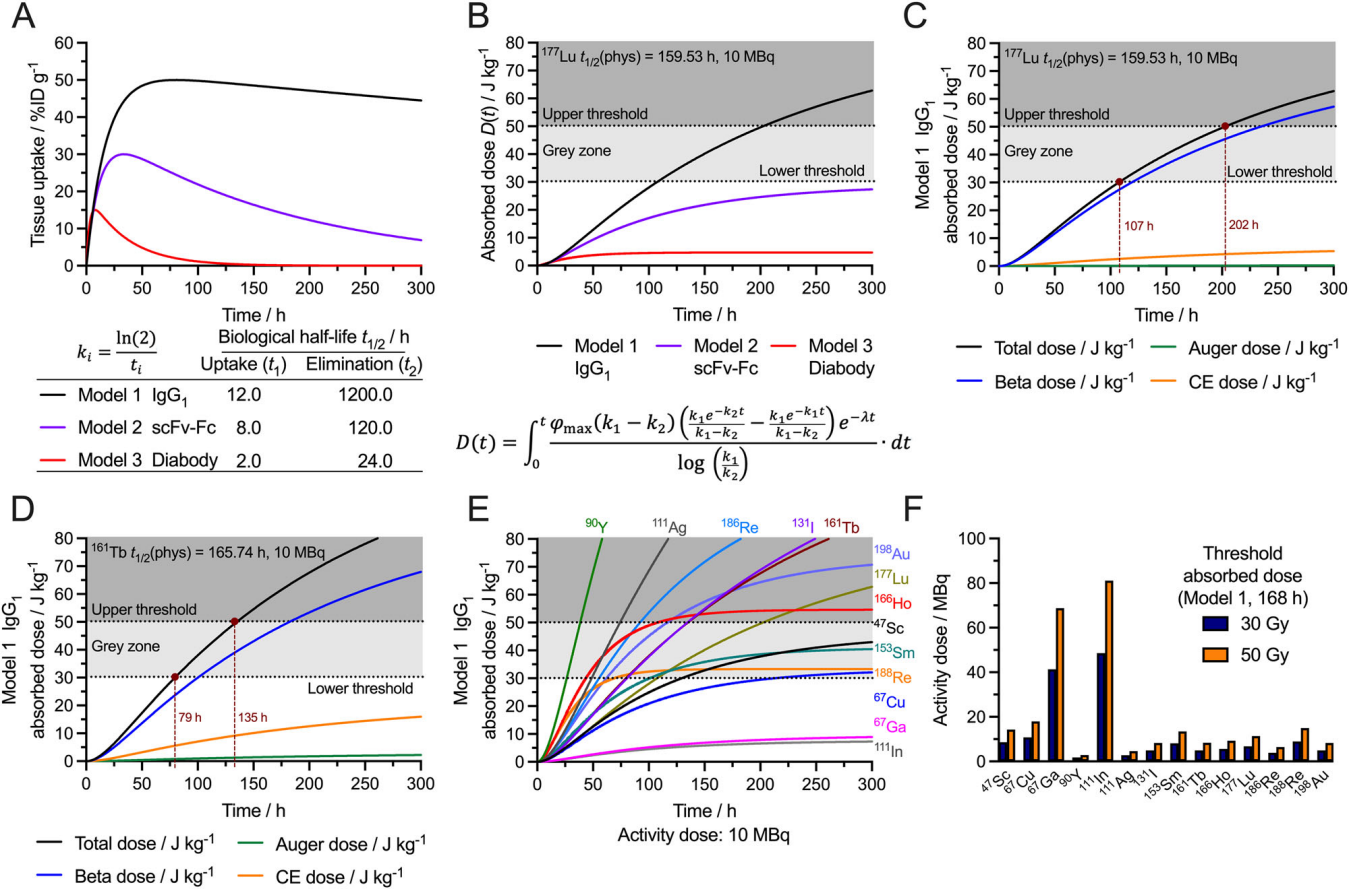
Modelling the pharmacokinetic profile and tissue absorbed dose (in J kg^−1^) for protein-based radiotracers. **A** Modelled tissue (for example, tumour) time-activity curves based on the experimental studies from Wu^48^ and co-workers in rodents (see Fig. 3B) showing profiles generated from typical uptake and elimination biological half-lives associated with Model 1: a full-length IgG_1_ (~150 kDa, black), Model 2: an scFv-Fc fragment (~100 kDa, purple) and Model 3: a diabody (~50 kDa, red). *DoseItRight*^©^ web-application: https://doseitright.streamlit.app/. **B** Plot of the calculated absorbed dose for ^177^Lu using pharmacokinetic models 1–3. **C** Deconvolution of the contributions of different particle emissions to the total absorbed dose for the example using ^177^Lu-Model 1. **D** An equivalent plot of the adsorbed dose for Model 1 using ^161^Tb. **E** An overlay showing the total absorbed dose calculated for Model 1 using 14 different therapeutic radionuclide. **F** Bar chart showing the activity required to achieve threshold absorbed dose of 30 Gy (blue bars) and 50 Gy (yellow bars) at an arbitrary time point of 168 h post-radiotracer administration for 14 different radionuclides.

Examples of three pharmacokinetic profiles plotting decay-corrected tissue activity (in %ID g^−1^) *versus* time are presented in Fig. 10A. The uptake and elimination half-lives for the three models shown were selected to mirror experimental data from Wu and co-workers (*vide supra* Fig. 3B). Model 1 replicates the distribution of a full-sized IgG_1_ (150 kDa, with tissue uptake *t*_1_ = 12 h and slow elimination *t*_2_ = 1200 h). Model 2 equates to an scFv-Fc (100 kDa fragment) and model 3 to a small diabody (50 kDa fragment).

Estimation of the dosimetric profile (absorbed dose versus time) for each combination of model and radionuclide uses a normalised Bateman equation with corrections for the user-defined *φ*_max_ and decay-correction of the chosen radionuclide (Fig. 10B). Note, *D*(*t*) is the cumulative absorbed dose in J kg^−1^ (Gy) at time *t* after radiotracer administration. Further deconvolution of the total (sum) absorbed dose in terms of individual contributions from the different decay modes and particles (α-particles, β-particles, conversion electrons (CE), and Auger electrons) is also presented for Model 1 with an administered dose of 10 MBq using either ^177^Lu (Fig. 10C) or ^161^Tb (Fig. 10D). The two threshold doses that act as boundaries for the light grey shaded area represent an upper bound at 50 Gy, which is the common target dose administered to tumours from external X-ray beam therapy and is usually considered lethal, and an arbitrary lower threshold at 30 Gy (which is ill-defined and must by identified empirically). The intermediate dose between these thresholds is the ‘grey zone’ where cell killing is likely to occur, but therapeutic responses may be incomplete. In our application, the user is free to define these dose thresholds, as well as the time required to reach a particular dose, to facilitate accurate estimation of the required administered activity. Interestingly, for the hypothetical comparison of radiotracers that display identical pharmacokinetics (Model 1), the use of an administered activity of 10 MBq of ^177^Lu attains a 30 Gy threshold in 107 h and 50 Gy in 202 h, whereas the use of ^161^Tb crosses the same dose thresholds in 79 h and 135 h, respectively. Comparison of the total dosimetric profiles for 14 different therapeutic radionuclides (using Model 1 and a 10 MBq administered dose) is shown in Fig. 10E. Supporting information Fig. S9 also shows a deconvolution of the total dose in terms of contributions from different particle emissions (α-particles, β-particles, conversion electrons (CE), and Auger electrons) for 14 different therapeutic radionuclides.

Finally, it is possible to predict how much activity of a given radionuclide is required to achieve a desired threshold dose at a user-defined time point. As an example, data presented in Fig. 10F shows a bar chart of the required administered activity to achieve either a 30 Gy or 50 Gy dose threshold at an arbitrary time point (here, set at 168 h) for the same 14 different therapeutic radionuclides using Model 1. In this case, using a radiolabelled IgG_1_ molecule that displays a pharmacokinetic profile consistent with Model 1 would require administering 11.4 MBq of ^177^Lu but only 8.4 MBq of ^161^Tb to achieve the same 50 Gy threshold at 168 h post-radiotracer injection.

In spite of the current limitations of the *DoseItRight*© application, the simulations provide a reliable way to estimate the absorbed dose, which facilitates the selection of reasonable administered doses of activity for designing radiotherapy experiments. In addition, the ability to choose different radionuclides allows for a rapid comparison of the expected dosimetric profile which also aids in experimental design. Notably, the tool is not restricted to pharmacokinetic profiles assigned to ‘tumour’ (this label is arbitrary) and if organ time-activity curves are available from pilot imaging data, dosimetry estimates can be performed for other tissues. We believe that this dosimetric tool will facilitate improved selection of activity doses in preclinical radiotherapy experiments which will be useful in designing head-to-head studies aimed at comparing the therapeutic effects of radiotracers with different pharmacokinetic profiles, or for radiotracers with similar pharmacokinetic profiles but carrying different radionuclides. Notably, the programme also functions with alpha particle emitters. Future work will expand the capabilities of *DoseItRight*© to allow dosimetric predictions of radionuclides that release radioactive daughters.

## Conclusion and future perspective

Radiotracer development is accelerating at an unprecedented pace. As scientists gain access to an increasingly large pool of diagnostic and therapeutic radionuclides, and as biologically active vectors allow the interrogation of disease biomarkers with unprecedented resolution and accuracy, new opportunities emerge to help diagnose and treat patients. Established concepts in radiopharmaceutical design have served the radiochemistry community very well for over 4 decades, but as we move toward radiotherapy, especially using high activity doses and radionuclides that cause high levels of ionising radiation damage, it is becoming increasingly important to refine the pharmacokinetic profile of radiopharmaceuticals with the simultaneous aims of retaining high and specific tumour uptake, reducing off-target exposure, and improving the therapeutic window. Chemistry plays a central role in solving the multifaceted challenges associated with controlling radiopharmaceutical pharmacokinetics and dosimetry profiles. Through innovative chemical design and the development of cleavable linker technologies, and an improved understanding of how radiopharmaceuticals interact with the body, we believe that future radiotheranostic compounds will be able to harness biochemical pathways to control radiopharmaceutical metabolism and excretion rates at the tissue-specific level.

## Supplementary information

Supplementary Information
