# Supplementary material for: New tactics in the design of theranostic radiotracers
Source: Npj Imaging. 2024 Aug 2;2:23. doi: 10.1038/s44303-024-00027-1 (PMC12118753; doi:10.1038/s44303-024-00027-1)
Supplement: Supplementary file 1 — Supplementary Information [file 44303_2024_27_MOESM1_ESM.pdf]

Supporting Information

New tactics in the design of theranostic radiotracers

Cesare Berton,[Δ] Simon Klingler,[Δ] Stanislav Prytuliak, and Jason P. Holland*

University of Zurich, Department of Chemistry, Winterthurerstrasse 190, CH-8057, Zurich, Switzerland

*** Corresponding Author:**

Prof. Dr Jason P. Holland

ORCID: orcid.org/0000-0002-0066-219X

Tel: +41-44-63-53990

E-mail: jason.holland@chem.uzh.ch

Website: www.hollandlab.org

[Δ] authors contributed equally.

User guide to the *DoseItRight*© dashboard

The program has been written in Python, the code is publicly available at the GitHub repository found at the link: https://github.com/cecebert/dosimetry_dashboard.

The dashboard is freely accessible at the link https://doseitright.streamlit.app/. The app is accessible on standard personal computers, smartphones, or tablets.

The authors accept no liability for any damages or errors that may occur in any form, from use of the application or the data generated.

Use of the application is free of charge but any publications involving the data should provide citation to this article and to the website of the application (CC-BY-NC).

This dashboard is designed for users without prior experience in simulations and coding. The following paragraphs provide a simple step-by-step tutorial.

1) User interface and first steps:

In the browser, enter the link https://doseitright.streamlit.app/ which directs to the landing page of the dashboard (Figure S1).

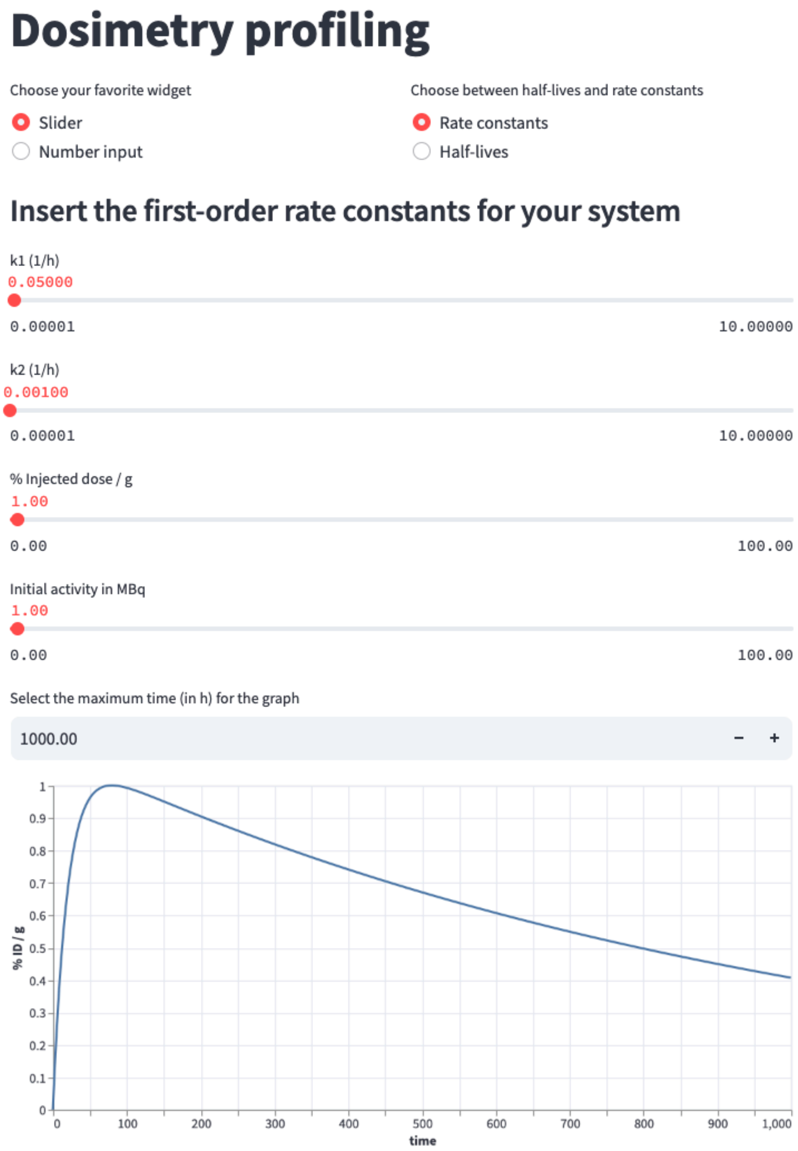

Figure S1. Landing page screenshot of the *DoseItRight*© dashboard.

In the top-right corner of the display, it is possible to tune various website settings like the theme (which can be alternated between light and dark), re-running of the calculation, printing of the current screen, and screencast recording (Figure S2).

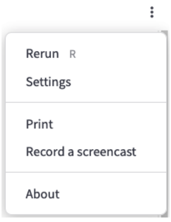

Figure S2. Settings are found in the top-right corner of the webpage.

2) Kinetic parameters and simulations

The first set of widgets are the selectors that define the simulation parameters used to simulate the kinetic profile (Figure S3). Simulation of the kinetic profile of tissue uptake (in units of %ID g^{-1}) requires user input of the rate constants for the uptake and elimination steps. These data can be input as either rate constants, k_1(uptake) / h^{-1} or k_2(elimination) / h^{-1}, or alternatively in mor intuitive values of time, t_1(uptake) / h or t_2(elimination) / h. A toggle button is used to switch between these input formats. A second toggle button allows the user to choose between the use of a slide bar to select the values of k or t, or a 'number input' for more precise control.

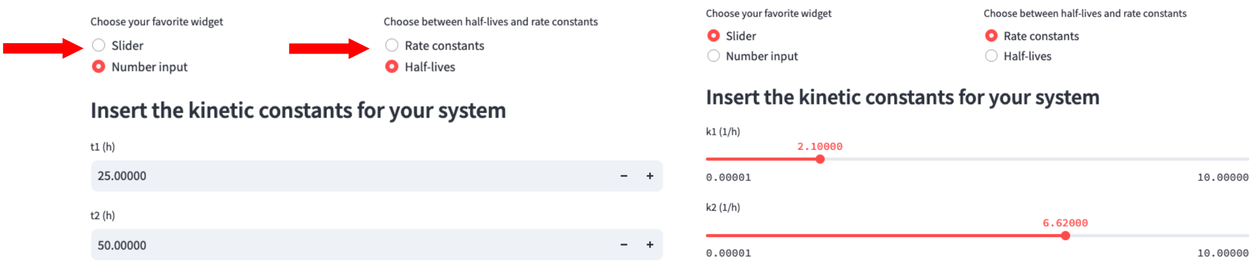

Figure S3. Interchangeable selection of the input formats by pressing buttons is easily accomplished in *DoseItRight*©. (Left panel) The number input and time t / h style. Pressing the buttons indicated with the red arrow, switches the display to the one shown in the (Right panel) where the rate constants are selected using a slider.

By default the kinetic profiles for 'tissue uptake' / %ID g^{-1} *versus* time (hours) are normalized to a maximum tissue uptake value of 1.00. This can edited by changing the value of the maximum percentage injected dose per gram (%ID g^{-1}), selecting a value that matches the experimentally measured peak uptake from pilot studies.

In addition, the use can select the initial activity of the radiotracer at administration (Initial activity in MBq) and the maximum time (in hours) for displaying the kinetic profile.

These input parameters are followed by a line chart that represents the time evolution of the tissue uptake in %ID g^{-1} of the tracer. Note, the selection of an initial activity other than 1.0 MBq (or a different radionuclide) has no impact on the pharmacokinetic profile (Figure S4) but does affect the calculated absorbed dose (see below).

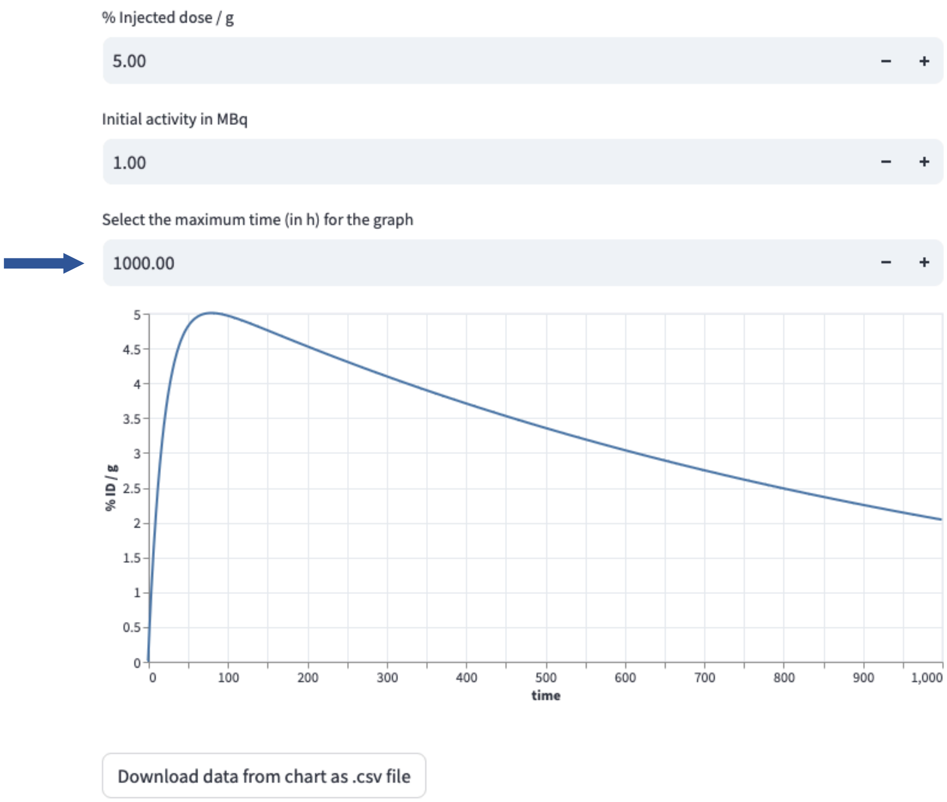

Figure S4. Chart of the time evolution of the tissue uptake (%ID g^{-1}) given the initial parameters and the span of the *x*-axis. Note: the *x*-axis span of the chart can be tuned by modifying the value in the correspondent box (blue arrow).

The contents of the graph are interactive, and it is possible to hover on the plotted line to obtain the corresponding coordinates and to download the data from the chart as a .csv file (which can be easily opened by using spreadsheet management programs or suitable software). The graph can be saved in .png and .svg formats as provided by the site.

3) Nuclide selection and activity *vs.* time

The *DoseItRight*© application creates a plot showing the activity present in the tissue of interest *versus* time (Figure S5). In effect, this is a decay correction of the kinetic chart shown in Figure S1, multiplied by a scaling factor to account for the maximum percentage injected dose per gram measured in the tissue. Decay-correction is related to the half-life of the radionuclide which is selected from a menu. The half-life and decay properties of each nuclide are obtained automatically from an internal database available with the Python library "*radioactivedecay*" found at the page https://radioactivedecay.github.io/.

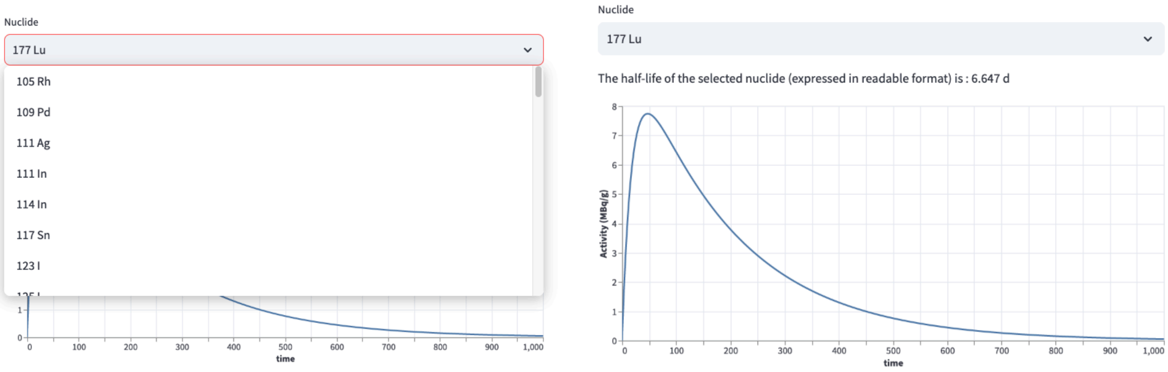

Figure S5. (Left) Drop-down menu for selecting the radionuclide of interest (including search capabilities). (Right) The correspondent activity (in MBq / g) versus time (in hours) plot. The plotted data are equivalent to the decay-corrected tissue activity curve.

4) Particle selection for dosimetry

Radionuclide decay is normally accompanied by a range of different particle emissions and data associated with the decay of the selected radionuclide is automatically extracted from the "*radioactivedecay*" Python library. The next selection allows the user to select particular particle emission types to either include or exclude them from the dosimetric calculation (Figure S6). Here, α = alpha-particles, β = beta-particles, Auger = Auger electron emission, CE = conversion electrons. By default, all reported emissions for a given radionuclide decay are included. If, for example, a radionuclide does not emit α-particles, this column is omitted and cannot be selected.

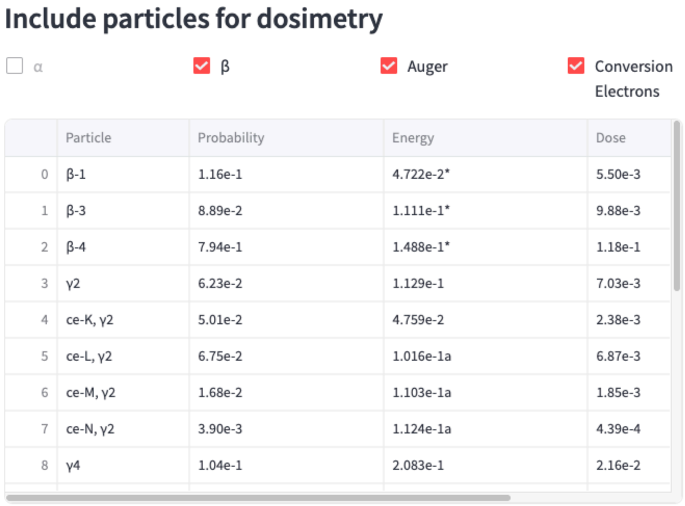

	Particle	Probability	Energy	Dose
0	β-1	1.16e-1	4.722e-2*	5.50e-3
1	β-3	8.89e-2	1.111e-1*	9.88e-3
2	β-4	7.94e-1	1.488e-1*	1.18e-1
3	γ2	6.23e-2	1.129e-1	7.03e-3
4	ce-K, γ2	5.01e-2	4.759e-2	2.38e-3
5	ce-L, γ2	6.75e-2	1.016e-1a	6.87e-3
6	ce-M, γ2	1.68e-2	1.103e-1a	1.85e-3
7	ce-N, γ2	3.90e-3	1.124e-1a	4.39e-4
8	γ4	1.04e-1	2.083e-1	2.16e-2

Figure S6. Particle selector for dosimetry calculations in *DoseItRight*©.

The particle table is provided for quick reference and contains decay information for the specific radionuclide including columns for the probability, energy (MeV), and dose (MeV Bq^{-1}) per particle. Note, only α-particles, β-particles, Auger electrons and conversion electrons are considered in the dosimetry and gamma rays (γ-rays) are excluded.

5) Total and particle absorbed dose *versus* time

After selection of the desired particles in section 4, it is possible to inspect numerically and graphically the results derived from the calculation of the absorbed dose (in Gy; equivalent to SI units of J kg^{-1}) deposited in the tissue of interest (Figure S7). Note, the calculation assumes that all energy is deposited in the tissue (no crossfire / bystander effects – see the main text for more detail).

Time-dependent dose per particle

	time	Beta Dose	Auger Dose	CE Dose	Sum
0	0	0	0	0	0
1	0.3191	0	0	0	0
2	0.6382	0.0021	0	0.0002	0.0023
3	0.9573	0.0084	0	0.0008	0.0092
4	1.2764	0.0187	0.0001	0.0018	0.0205
5	1.5954	0.0331	0.0001	0.0031	0.0363
6	1.9145	0.0513	0.0002	0.0048	0.0564
7	2.2336	0.0735	0.0003	0.0069	0.0807
8	2.5527	0.0994	0.0004	0.0094	0.1092
9	2.8718	0.129	0.0005	0.0121	0.1417

Required total dose

60.00 − +

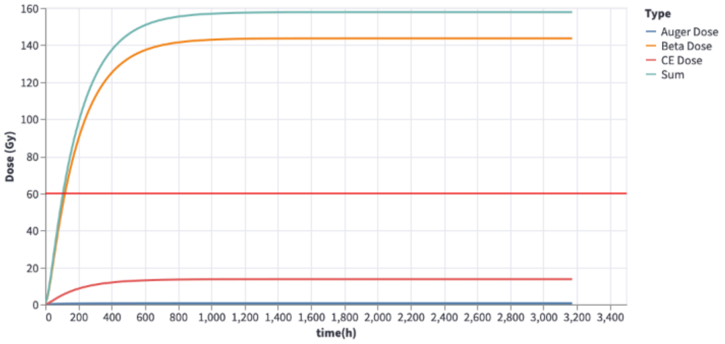

The time for delivering the total required dose of 60.00 Gy is 111.68 hours

Download data from chart as .csv file

Figure S7. Time dependency of the absorbed dose (J kg^{-1}) dose grouped by particle type and also represented as sum. Data are tabulated (top), and also displayed as an interactive chart (bottom). The chart features an adjustable threshold for quick estimation of the time required to reach a certain absorbed dose with the given combination of kinetic parameters, radionuclide, and injected dose.

6) Time for dose estimator

The last feature of *DoseItRight*© is a tool which allows the calculation of how much activity must be administered to reach a user-defined absorbed dose at a user-defined time.

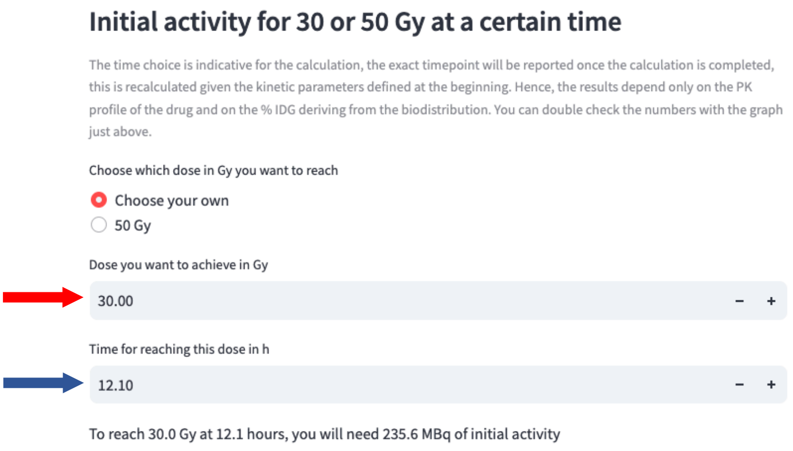

Figure S8. Selection of a desired dose threshold and time point. This feature allows the user to determine how much initial activity (MBq) of the radiotracer must administered to achieve a threshold absorbed dose in a user-defined time. The widget is useful for estimating initial administered activities and for assisting in the design of fractionated dosing regimen, particularly for radiotracers that exhibit rapid uptake and elimination pharmacokinetics, or for those that are constructed with radionuclide that have short half-lives.

DoseItRight© calculates the initial activity of radiotracer to be administered to reach the input dose within the input time. In the case of Figure S8, using the specific kinetics defined in steps 1-3, it is possible to reach 30 Gy in 12.1 h by administering 235.6 MBq of radiopharmaceutical to the patient.

7) Selected examples

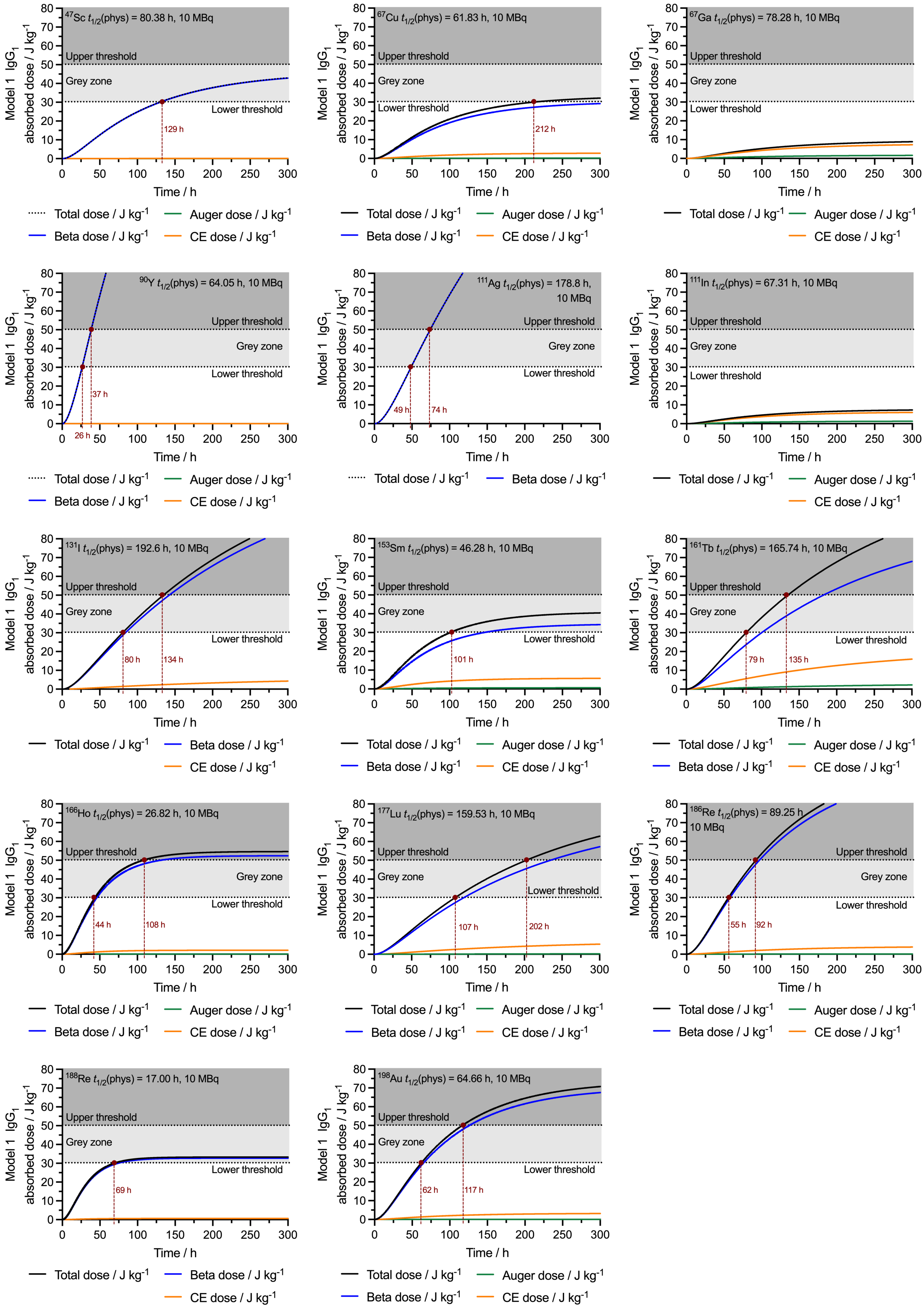

Figure S9. Plots of the calculated absorbed dose time *versus* time for a series of selected therapeutic radionuclides based on the pharmacokinetic profile of model 1 (see main text). Here, the models have identical kinetic profile but the plots illustrate how the absorbed dose changes with the choice of radionuclide. In addition, the data show how, for a given radionuclide, different particle emissions contribute to the total absorbed dose.
